# *Arabidopsis thaliana* zinc accumulation in leaf trichomes is correlated with zinc concentration in leaves

**DOI:** 10.1038/s41598-021-84508-y

**Published:** 2021-03-05

**Authors:** Felipe K. Ricachenevsky, Tracy Punshon, David E. Salt, Janette P. Fett, Mary Lou Guerinot

**Affiliations:** 1 Programa de Pós-Graduação em Biologia Celular e Molecular, Centro de Biotecnologia, Universidade Federal do Rio Grande Do Sul, Porto Alegre, Brazil; 2 Departamento de Botânica, Instituto de Biociências, Universidade Federal do Rio Grande do Sul, Av. Bento Gonçalves, Porto Alegre, RS 9500 Brazil; 3 Department of Biological Sciences, Life Sciences Center, Dartmouth College, 78 College St, Hanover, NH 03755 USA; 4 Future Food Beacon of Excellence and the School of Biosciences, University of Nottingham, Nottingham, LE12 5RD UK

**Keywords:** Plant physiology, Natural variation in plants, Plant genetics

## Abstract

Zinc (Zn) is a key micronutrient for plants and animals, and understanding Zn homeostasis in plants can improve both agriculture and human health. While root Zn transporters in plant model species have been characterized in detail, comparatively little is known about shoot processes controlling Zn concentrations and spatial distribution. Previous work showed that Zn hyperaccumulator species such as *Arabidopsis halleri* accumulate Zn and other metals in leaf trichomes. To date there is no systematic study regarding Zn accumulation in the trichomes of the non-accumulating, genetic model species *A. thaliana*. Here, we used Synchrotron X-Ray Fluorescence mapping to show that Zn accumulates at the base of trichomes of *A. thaliana*. Using transgenic and natural accessions of *A thaliana* that vary in bulk leaf Zn concentration, we demonstrate that higher leaf Zn increases total Zn found at the base of trichome cells. Our data indicates that Zn accumulation in trichomes is a function of the Zn status of the plant, and provides the basis for future studies on a genetically tractable plant species to understand the molecular steps involved in Zn spatial distribution in leaves.

## Introduction

Zinc (Zn) is an essential micronutrient, serving as a cofactor for many enzymes and transcription factors^1^. An estimated 8% of the *Arabidopsis thaliana* proteome binds to Zn^2^. In humans, Zn deficiency is the second most widespread nutritional deficiency after iron (Fe) deficiency, with an estimated 25% of the population at risk of low Zn intake, especially when the diet is composed mostly of cereal grains^3,4^. Since plants are the primary source of Zn entry into the food chain, understanding Zn homeostasis is essential to produce plants that accumulate more Zn in their edible tissues^5,6^.

The genetic regulation of Zn uptake, distribution, detoxification and storage in *A. thaliana* has been functionally characterized^6,7^. Most of our knowledge is focused on root control of Zn acquisition and Zn excess detoxification. Primary Zn uptake from the soil is likely performed by members of the ZIP (Zinc-regulated/Iron-regulated transporter Protein) family^8–10^. In rice, OsZIP9 was recently described as responsible for Zn acquisition from the rhizosphere^11–13^. The ZIP transporters were the first Fe and Zn transporters characterized in plants, and have been shown to transport Fe, Zn and other divalent cations in several plant species^14–17^. The *A. thaliana* bZIP19 and bZIP23 transcription factors control the Zn deficiency response, in which ZIP transporters are upregulated^8,18,19^. AtZIP4 and AtZIP9, two direct targets of bZIP19 and bZIP23, are up regulated in roots in response to a local Zn deficiency signal. On the other hand, AtMTP2 (Metal Tolerance Protein 2), an endoplasmic reticulum (ER)-localized Zn transporter, is also up regulated in roots, but in response to a shoot-derived Zn deficiency signal^10^. Other members of this family, vacuolar transporters such as AtMTP1^20,21^ and AtMTP3, detoxify excess Zn by sequestering it into the vacuole. ZIF-Like (Zinc-Induced Facilitator) family transporters may be involved in Zn tolerance, either through transport of Zn bound to nicotianamine (AtZIF1^22^) or direct Zn sequestration (AtZIF2^23^) in vacuoles. Moreover, Zn efflux transporters such as AtHMA2 and AtHMA4 (Heavy Metal-Associated transporters) perform Zn loading from the root symplast into the xylem for long distance transport^24^, and are also involved in Zn loading in seeds^25^.

Zn accumulation and storage in shoots, on the other hand, is not well understood. Previous work in Zn hyperaccumulator species *A. halleri* and *Nocceae caerulescens*^26–28^ which are close relatives of *A. thaliana*, show how critical Zn homeostasis genes are in establishing their remarkable tolerance to Zn levels that are lethal to other species. Both *A. halleri* and *N. caerulescens* rely on multiple copies of genes such as *MTP1* and *HMA4* for their Zn hyperaccumulation phenotypes^29–31^. Previous work has shown that Zn accumulation might be related to herbivory deterrence^32,33^. *A. halleri* accumulates high concentration of Zn in its trichomes. This accumulation occurs specifically at the base of tricomes as a narrow ring^34–36^ Elements such as cadmium (Cd), for which *A. halleri* is also hypertolerant, also accumulate in trichomes^37^. Because the hyperaccumulator/hypertolerant species *A. halleri* and the non-hyperaccumulator *A. lyrata* both accumulate Zn and Cd in trichomes^34,35,37^ in a similar pattern, it seems unlikely that this is a hyperaccumulation mechanism.

*A. thaliana* non-glandular tricomes are derived from epidermal cells that undergo endoreduplication, which consists of replication of the genome without mitosis, and results in increased cell size^38^. These trichomes differ from the secretory trichomes, such as those found in tobacco, which secrete Cd and Zn^39,40^. Despite not having a secretory function, non-glandular trichomes are likely hotspots for metal accumulation^41,42^. Cd has been detected in *A. thaliana* non-glandular trichomes^43,44^. Non-glandular trichomes of sunflower (*Helianthus annuus*) accumulate Mn when the metal is in excess in the growth medium^41^, while Zn accumulates rapidly at the trichome base when Zn is sprayed on leaves^45^, a phenomenon also observed in soybean^46^, suggesting that trichomes are a sink for metals both from foliar application or the transpiration stream. Despite the work on *A. lyrata* and *A. halleri*, little is known about metal accumulation in the trichomes of the model species *A. thaliana*.

Our aim in this study was to understand how Zn availability affects *A. thaliana* Zn accumulation in trichomes and in leaves. To do this we conducted elemental mapping using synchrotron X-ray fluorescence (SXRF) of both *A. thaliana* natural variants and transgenic plants provided with a variety of Zn concentrations in the growth medium. Non-glandular trichomes accumulated more Zn as leaf Zn concentrations increased and Zn accumulation at the base of the trichome was observed. Our results suggest that plants may actively change the partitioning of Zn to trichomes in response to leaf Zn supply.

## Results

### OsZIP7 constitutive expression leads to altered Zn distribution in leaves

**Figure 1:**
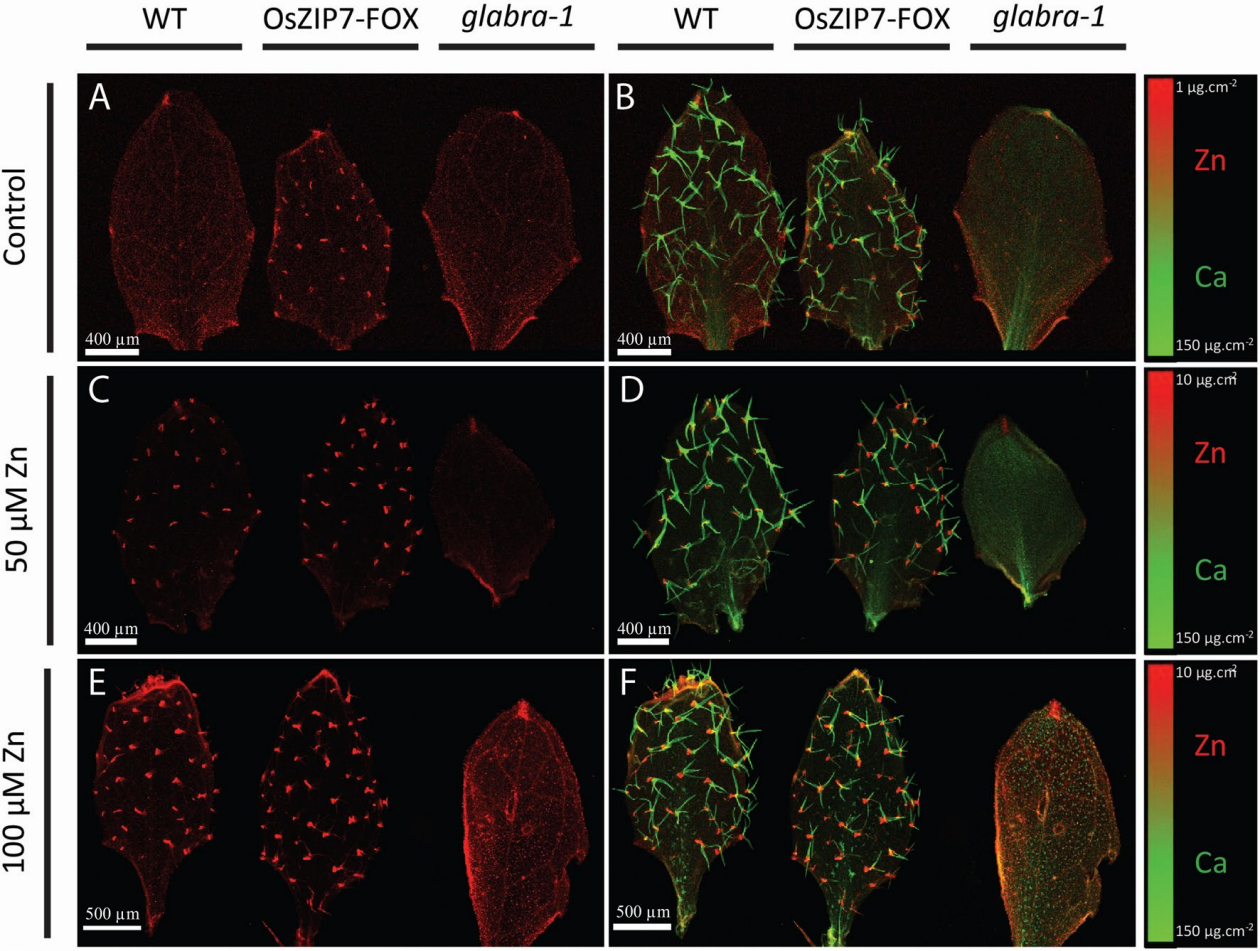
Elemental maps of Col-0, OsZIP7-OE and the *glabrous-1* mutant leaves. Maps show Zn localization (in red) in leaves of plants grown under 50 nM Zn (**A**), 50 μM Zn (**C**) and 100 μM Zn (**E**), and Zn and Ca (in green) overlay in leaves from plants grown under 50 nM Zn (**B**), 50 μM Zn (**D**) and 100 μM Zn (**F**).

We previously showed that *OsZIP7* expression under the control of 35S promoter in *A. thaliana* leads to increased Zn concentration in leaves^16^. Here, to investigate alterations in Zn localization, we used two-dimensional SXRF mapping of leaves of wild type (WT) and *OsZIP7*-expressing plants (hereafter OsZIP7-FOX) to identify possible changes in Zn distribution. This technique provides information about all Zn regardless of chemical speciation. Leaves of plants grown under 50 nM Zn (our control condition) showed Zn evenly distributed throughout the leaf, with higher concentrations in hydathodes and closer to the petiole detachment site (Fig. 1A). In leaves of OsZIP7-FOX, Zn was found highly concentrated in small, punctuated areas at the leaf surface (Fig. 1A). When Zn localization was overlaid with localization of Ca, which is known to accumulate in leaf trichome papillae^47^, it was clear that Zn was accumulating at the base of trichomes in leaves of OsZIP7-FOX plants, a distribution that was not observed in WT (Fig. 1B). Leaves from *glabra-1* mutant plants^48^, which lack trichomes, showed a uniform Zn distribution across the leaf surface similar to WT when plants are grown under 50 nM Zn (Fig. 1A,B). However, when plants were grown on media supplemented with 50 µM or 100 µM Zn, the punctate pattern of Zn distribution associated with the base of trichomes was observed in both WT and OsZIP7-FOX leaves (Fig. 1C–F) whereas leaves of the trichome-less *glabra-1* mutant showed evenly-distributed Zn and Ca. Such high Zn concentrations, although not typically seen in the environment, were used to maximize Zn availability to plants and therefore increase the likelihood of Zn accumulation in trichomes. These results confirm that Zn is accumulating at the base of trichomes, as observed for *A. lyrata* and *A. halleri*^34,36^.

**Figure 2:**
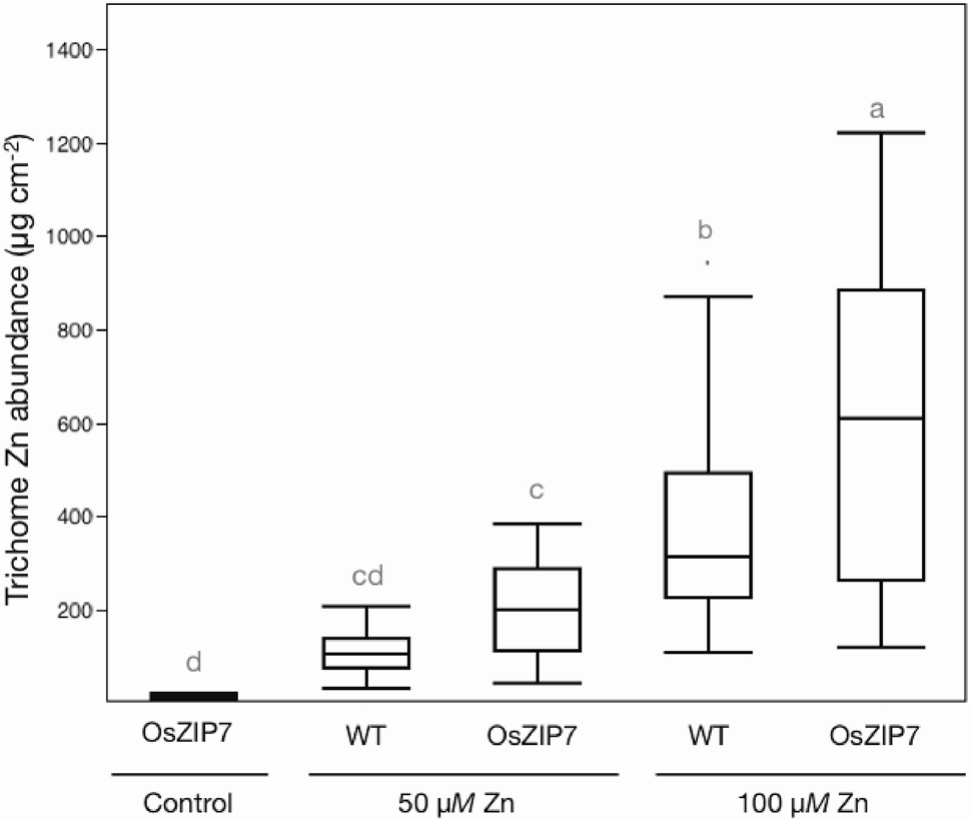
Total Zn abundance in individual trichomes. Total Zn per trichome was estimated using Region of Interest (ROI) analyses. All trichomes in each leaf showing Zn accumulation in the maps were included in the analyses (n = 30–32). Significant differences detected by one-way ANOVA and Tukey HSD are represented by different lower case letters above the boxplots.

User-defined region of interest (ROI) analysis allowed us to determine total Zn per trichome from mapping data collected via SXRF. The OsZIP7-FOX leaf grown under 50 nM Zn had the lowest total Zn per trichome, considering the maps in which Zn in trichomes was observed (i.e., excludes WT Col-0 leaves under 50 nM Zn conditions; Fig. 2). In leaves of plants growing at 50 µM Zn, total Zn per trichome was higher in both WT and OsZIP7-FOX compared to OsZIP7-FOX under 50 nM Zn. The total Zn per trichome in WT and OsZIP7-FOX grown with 50 µM Zn were not significantly different (Fig. 2). However, comparing WT and OsZIP7-FOX leaves from plants grown with 100 µM Zn, OsZIP7-FOX trichomes had clearly higher total Zn per trichome (Fig. 2). We showed previously that OsZIP7-FOX lines accumulate higher Zn concentration in their leaves compared to WT under these conditions^16^. Our data indicate that OsZIP7 expression in *A. thaliana*, which leads to increased accumulation of Zn in leaves, also leads to accumulation of Zn at the base of trichomes.

### Trichomes accumulate Zn in a ring around the base in *Arabidopsis thaliana*

**Figure 3:**
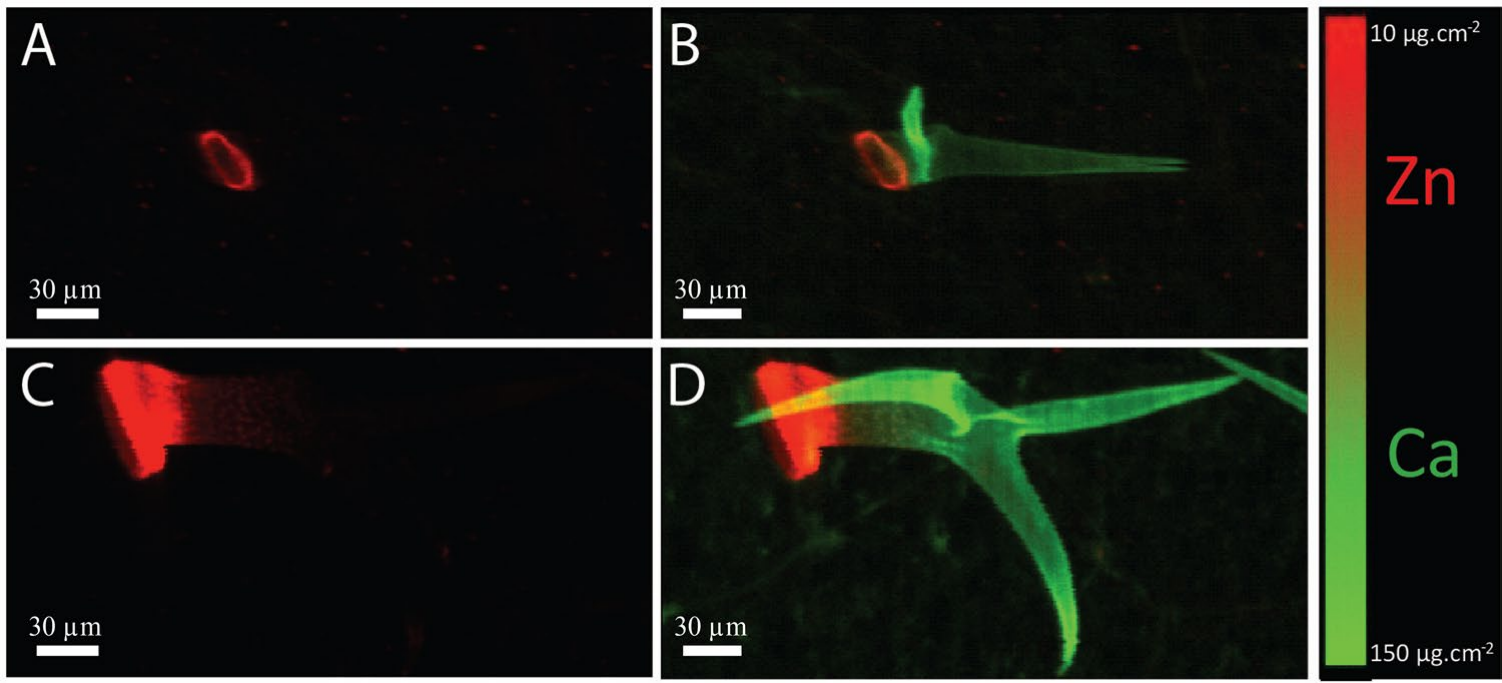
High-resolution elemental maps of individual fresh hydrated trichomes. Maps show Zn (in red) in trichomes of Col-0 (**A**) and OsZIP7-OE (**C**), and Zn and Ca (in green) localization overlay of Col-0 (**B**) and OsZIP7-OE (**D**).

To gain more information on the nature of the characteristic pattern of Zn accumulation at the base of trichomes, we used higher-resolution SXRF mapping of fresh trichomes. Plants grown at 50 µM Zn were chosen because both WT and OsZIP7-FOX leaves accumulate Zn in trichomes under these conditions (Fig. 1C,D), and this concentration was not toxic to *A. thaliana* plants under our experimental conditions^16^. Zn localization maps of WT leaves showed a ring-shaped pattern around the base of the trichome (Fig. 3A,B). In the mapped trichome of the OsZIP7-FOX plant, the Zn ring was thicker than in the mapped trichome of the WT plant, appearing to increase its domain farther away from the trichome base and up into the stalk (Fig. 3C,D). Because these are individual trichomes, and the median total Zn per trichome from plants cultivated under these conditions are not statistically different (Fig. 2), the distinct Zn distribution pattern observed is likely found in trichomes of both WT and OsZIP7 plants. Therefore, our data show that Zn accumulation at the base of the trichome occurs in a ring shape, which can vary in thickness depending on Zn concentration.

### Variations in leaf Zn concentration influence accumulation at the base of trichomes in *A. thaliana* natural accessions

**Figure 4:**
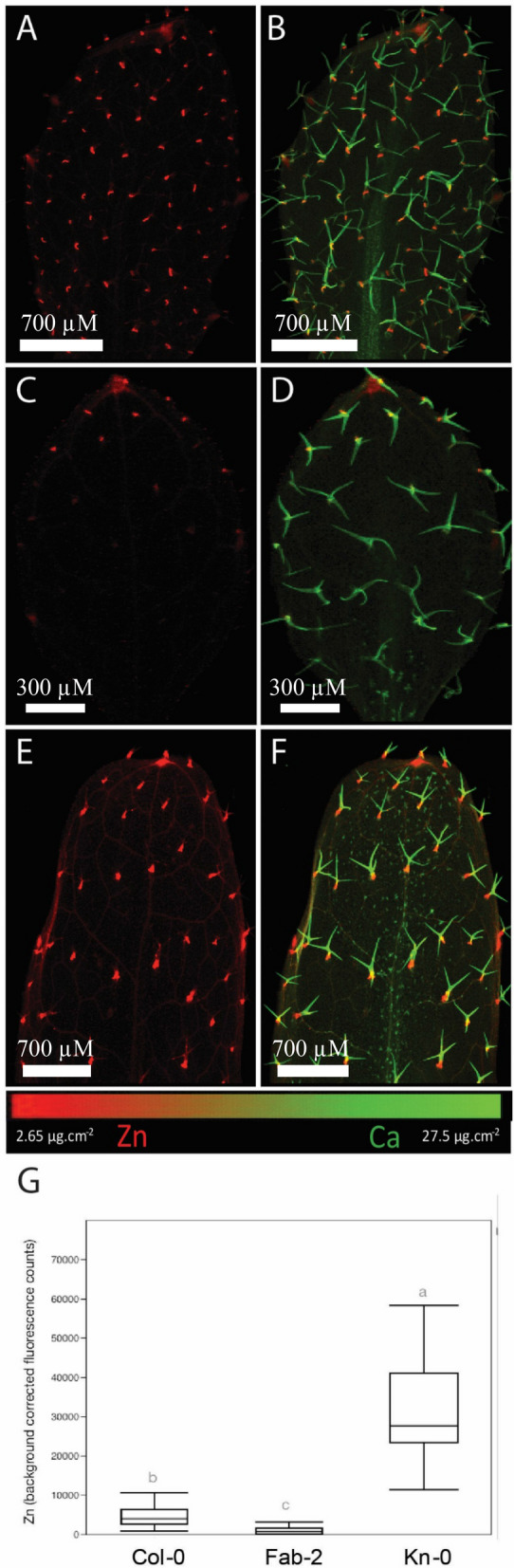
Total Zn per trichome of *A. thaliana* natural accessions with contrasting bulk leaf Zn concentrations. Total Zn abundance in trichomes was quantified using Region of Interest (ROI) analysis of leaves. All trichomes in each leaf presented in the maps were included in the analyses (n = 25–61). Significant differences by one-way ANOVA and Tukey HSD are shown. Panels show Col-0 leaf Zn (**A**) and Zn and Ca (**B**); Fab-2 leaf Zn (**C**) and Zn and Ca (**D**); Kn-0 leaf Zn (**E**) and Zn and Ca (**F**) localization. All plants were cultivated under 50 µM Zn, and the 7th true leaf was analyzed.

The first part of this study showed that *OsZIP7* expression in *A. thaliana* led to increased Zn accumulation at the base of trichomes (Figs. 1, 2, 3). We then asked whether the ability to accumulate different Zn concentrations in leaves would affect Zn in trichomes. To answer this question, we used Zn concentrations data from leaves of 349 *A. thaliana* natural accessions^49^ and selected a subset of accessions with high and low Zn concentrations (Table 1). We mapped leaves of the accessions with the highest and the lowest Zn concentrations, Kn-0 and Fab-2, respectively (Fig. 4, Table 1) derived from plants cultivated with 50 µM Zn. Fab-2 harbors a loss of function allele of *AtHMA4*, which decreases Zn translocation from roots to leaves^50^. Col-0 was included as the reference accession. Fab-2 leaves had several trichomes without Zn, and others with very low Zn fluorescence (Fig. 4C, D). Conversely, Kn-0 showed higher Zn fluorescence in its trichomes compared to Col-0 (Fig. 4A,B,E,F). ROI analyses of these maps showed that Kn-0 had higher total Zn per trichome, whereas Fab-2 trichomes showed lower total Zn per trichome, compared to the reference accession Col-0 (Fig. 4G). These data suggest that increased levels of Zn in leaves are correlated with increased Zn accumulation at the base of trichomes.

**Table 1:** Natural accessions of *A. thaliana* used in this work.

Group	Accession	Zn concentration (µg/g)^a^
Low Zn accessions	**Fab-2**	41.15
	**Rev-2**	70.44
	**Pn-0**	78.6
	**Ste-3**	79.31
	TAD 01	79.51
	**T1060**	80.03
	**Shahdara**	84.3
	Mc-0	84.54
	Wag-3	85.13
	**TOU-E-11**	87.79
Reference accession	**Col-0**	131.92
High Zn accessions	**ZdrI 2-25**	178.39
	**Ull2-5**	181.23
	**Ob-1**	182.69
	**PHW-13**	196.65
	Ors-2	196.76
	**NC-6**	201.48
	**Uod-7**	205.2
	**Na-1**	205.38
	Sav-0	238.5
	**Kn-0**	254.53

The ten highest and ten lowest Zn concentration in leaves are shown, from the 349 accessions found at the Ionomics Atlas. Accessions used are in bold and underlined.

^a^Data generated by ICP-MS and downloaded from the ionomics Atlas (http://www.ionomicshub.org/ionomicsatlas/).

**Figure 5:**
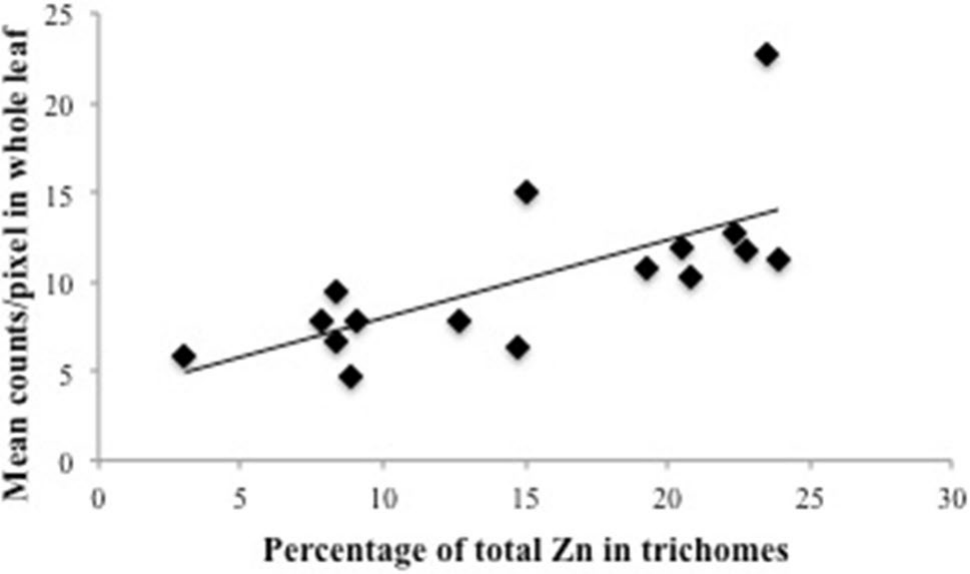
Zn accumulation in trichomes increases with Zn in leaves. Correlation of the percentage of the total Zn in trichomes with mean Zn counts in whole leaf of 16 *A. thaliana* accessions. All plants were cultivated under 50 µM Zn, and the 7th true leaf was analyzed.

In addition to Col-0, Kn-0 and Fab-2, we mapped other 13 accessions with contrasting leaf Zn concentrations for a total of 16 accessions (Table 1), also cultivated under 50 µM Zn. These accessions span the high and low ranges of Zn distribution in the iHUB, available at www.ionomicshub.org, allowing us to explore wide natural variation in leaf Zn concentration and its relationship to trichome Zn accumulation. Using ROI analyses, we determined the (1) mean count per pixel (a proxy for Zn concentration) per leaf; (2) percentage of total Zn sequestered within trichomes; and (3) percentage of Zn not associated with trichomes (Fig. 5).

Next, we compared the mean fluorescence counts per pixel with the percentage of total Zn found in trichomes, for each accession (Fig. 5). We found a positive correlation (R = 0.6871, *p* = 0.003) between the two variables, and consequently a negative, inverse correlation of Zn abundance not in trichomes (i.e., elsewhere in the leaf surface) and mean count per pixel. These data support the observations that *A. thaliana* accessions with higher leaf Zn concentrations accumulate a higher percentage of Zn at the base of trichomes, with a lower percentage elsewhere in the leaf. We therefore propose that Zn accumulation at the base of trichomes increases with increased Zn accumulation in the whole leaf, indicating trichomes may be an important Zn allocation site in leaves with high Zn concentrations.

## Discussion

### Zn accumulates at the base of trichomes in *A. thaliana*

Here we have demonstrated that *A. thaliana* accumulates Zn at the base of the trichome. In several plant species, trichomes are common sites of excess metal accumulation, including Pb, Zn and Cd in *Nicotiana tabacum*^39,40^, Cd in *Brassica juncea*^51^, Mn in *Helianthus annuus*^41^ and Ni in the hyperaccumulator species *Alyssum lesbiacum*^52^. In the Zn hyperaccumulator/hypertolerant *A. halleri*, the base of trichomes accumulates the highest concentrations of Zn in leaves^34,36^. Despite being regions of high accumulation, trichomes do not account for the majority of Zn in leaves: a comparison with the non-hyperaccumulator *A. lyrata* showed that Zn in trichomes of *A. halleri* accounts for 10% of the total, while *A. lyrata* trichomes account for 20% of the total^35^. The data for *A. lyrata* agrees with our findings in *A. thaliana*, another non-hyperaccumulator, with Zn accumulation in trichomes ranging from 4 to 23% (Fig. 5).

Our data show clear localization of Zn in trichome cells. Zn localization in the trichome cell itself is quite obvious in *A. halleri*, as the narrow Zn ring is localized more distal to the base, on the trichome stalk^34–36^. In *A. lesbiacum*, Ni has a similar distribution along the stalk, and the authors suggested that Ni could be stored inside vacuoles^52^. However, our mapping data from intact trichomes of WT and OsZIP7-FOX leaves showed Zn in a ring shape at the base and continuing up into the trichome stalk (Fig. 3), observations which are more consistent with an extracellular localization, because a vacuolar localization would presumably fill the trichome cell. In *A. halleri* trichomes, Zn accumulates in a small compartment at its base. Although in roots of *A. halleri* Zn precipitates in the apoplast, in trichomes both the chemical form and subcellular localization of Zn remain to be determined. Zn might be in a soluble form with O-donors such as citrate, or found in a precipitated solid form as Zn oxides given the high Zn concentration (> 1 M when plants are treated with excess Zn)^34^.

Previous work showed that Cd associated with trichomes was predominantly bound to oxygen (O) and nitrogen ligands in non-hyperaccumulators *A. thaliana* and *A. lyrata*, and in the hyperaccumulator *A. halleri*^37,53,54^. Cd may be associated with the cell wall in trichomes, bound to O ligands^37,54^. Similarly, in *A. halleri*, Zn also binds mainly to carboxyl and/or hydroxyl groups^55^. Phosphate, thiol and silanol groups were excluded as potential Zn ligands^35^. Interestingly, the non-hyperaccumulator *A. lyrata* showed more Zn bound to cell wall (40% of the Zn in trichomes) compared to hyperaccumulator *A. halleri* (20%)^35^. Recently a new structure, the Ortmannian ring, was described in *A. thaliana* trichomes^56^. Ortmannian ring formation is dependent on the EXO70H4 exocyst subunit and is a callose-rich secondary cell wall layer, localized between the basal and apical regions of the trichome stalk. Loss-of-function *exo70h4* plants showed no callose ring accumulation. Strikingly, WT and *exo70h4* differed in their ability to accumulate Cu in trichomes, with WT plants showing Cu accumulation at the base of the trichome, while *exo70h4* plants, which lack the Ortmannian ring, contain no Cu in the same region^56^. The data strongly suggest that the Ortmannian ring is involved in metal localization in trichomes. Therefore, Zn may co-localize with the Ortmannian ring, or its distribution is being limited by it. Further investigations are needed to clarify this. Moreover, experiments to fully characterize Zn subcellular localization in *A. thaliana* trichomes and its speciation and coordination environment should be performed in the future.

### Physiological significance of Zn accumulation in trichomes

We found that natural accessions of *A. thaliana* with higher whole leaf Zn have increased amounts of Zn in their trichomes (Figs. 4, 5). When comparing the percentage of Zn in trichomes with whole leaf total Zn, we found that accessions with higher Zn concentrations in whole leaves have an increased percentage of Zn in trichomes (Fig. 5). This leads us to hypothesize that trichome Zn accumulation might have a role in metal detoxification by providing a location for metal sequestration in *A. thaliana*.

Metal accumulation in trichomes has been shown before. In a previous study, four crop species were analyzed for Mn tolerance: sunflower (*Helianthus annuus*), white lupin (*Lupinus albus*), narrow-leafed lupin (*Lupin angustifolius*) and soybean (*Glycine max*). All but soybean could tolerate 100 µM Mn without showing toxicity symptoms^41^. Differently from the other Mn tolerant species, sunflower non-glandular trichomes accumulated Mn at the base, while Ca was distributed along the trichome length, resembling the pattern we observed in *A. thaliana* non-glandular trichomes with Zn and Ca. Fluorescence-XANES indicated that 66% of the Mn present at the base of the trichome is in the form of manganite [Mn(III)]^41^. The authors suggest that Mn is translocated from the apoplast to trichomes and then oxidized to manganite, thus preventing Mn accumulation in the cytoplasm and cell wall in leaf cells^41^. In the Ni-hyperaccumulator *Alyssum murale*, excess Mn accumulated in trichomes, where it was associated with phosphorous (P)^57^. It is possible that Zn in *A. thaliana* trichomes is being transported to the trichome apoplast directly via the transpiration stream, as has been proposed for Mn^41^.

*A. halleri* and *A. lyrata* (a non-hyperaccumulator) were found to accumulate Zn at the tricome base^34–37^. Zn accumulates to a higher extent in the mesophyll in *A. halleri* compared to veins, whereas the opposite is found in *A. lyrata*^35^. Another study observed that Zn concentration in mesophyll cells of *A. halleri* increased 30-fold upon exposure to high Zn, whereas Zn concentration in trichomes increased only 3-fold^34^. These results indicate that, despite their high Zn accumulation, trichomes are not important for Zn hyperaccumulation/hypertolerance in *A. halleri*. In tobacco (*Nicotiana tabacum*), the role of trichomes in heavy metal excretion is well documented: Zn and Cd are secreted from trichome tips as crystals^39,40,58^. However, these glandular, multicellular trichomes are very different from the unicellular, non-glandular trichomes found in *A. thaliana*^59^.

Cd and Mn accumulated at the base of *A. thaliana* trichomes^43,54^. Interestingly, *A. thaliana* transgenic lines that accumulate varying concentrations of Cd showed trichome metal accumulation varying in a similar way: more Cd in leaves resulted in more Cd in trichomes^43^. This is consistent with what we observed here for Zn in trichomes using 16 different accessions (Figs. 4, 5). Upon exposure to Cd, *A. halleri* accumulated Cd first in trichomes, and only later in other leaf tissues^44^, which suggests that trichome cells might be relevant for short-term response to metal excess, whereas leaves hyperaccumulation/hypertolerance becomes necessary in prolonged exposure. Likewise, *A. lyrata,* a non-hyperaccumulator, has more Zn in trichomes (20%) than in the hyperaccumulator *A. halleri* (10%)^35^. Thus, trichome metal sequestration and possibly detoxification might be more important in non-tolerant and non-accumulators *A. thaliana* and *A. lyrata* than in a metal hyperaccumulator, hypertolerant species.

Transgenic plants or natural accessions with increased Zn accumulation in leaves showed a higher percentage of Zn in trichomes. Therefore, our findings support the idea that Zn found in trichomes increases linearly with higher Zn leaf concentrations (Fig. 5). In *B. juncea*, experiments where mass flow is decreased by application of abscisic acid (ABA) to induce stomata closure, Cd accumulation in leaves is dependent upon transpiration, although root uptake is not affected^51^. This would indicate that Cd accumulation in trichomes is dependent on transpiration rate. However, our data shows that the amount of Zn found in trichomes change depending on how much Zn a leaf accumulates, which suggests an active mechanism for Zn accumulation in trichomes. Whether this mechanism involves cell wall modifications or symplast transport remains to be answered. Moreover, experiments addressing possible changes in trichome density and/or development under varying Zn (and other metal) concentrations should be key to unravel a possible function of these cells in tolerance or accumulation of Zn and other metals.

## Conclusion

Our work provides evidence for Zn accumulation in trichomes of *A. thaliana*, a genetically tractable model species, allowing exploration of the functional role of Zn distribution in trichomes and its relevance to leaf function. We also demonstrate that Zn accumulation in trichomes changes depending on Zn concentration in leaves, suggesting that plants might actively control this process to some extent. Future work should focus on the importance of such distribution in leaves and how it is molecularly controlled.

## Materials and methods

### Plant materials and growth conditions

All seeds from accessions used in this work were requested from the Arabidopsis Biological Resource Center (ABRC; https://abrc.osu.edu/). OsZIP7-OE lines used were previously described^16^.

For growth in axenic conditions, we performed experiments as described^16^, with minimal changes. Briefly, seeds were sterilized for 15 min in 25% NaOH and 0.05% SDS, washed 5 times in sterile H_2_O and stratified at 4 °C for three days. Sterile 0.1% agar was used to suspend seeds, which were sown using a pipette onto plates made with full strength Gamborg’s B5 media plus vitamins, 1 mM MES (2-(N-morpholino)ethanesulfonic acid), 2% sucrose and 0.6% agar. After five days, seedlings were transferred to minimal media containing 2 mM MES, 2 mM Ca(NO_3_)_2_.4H_2_O, 0.75 mM K_2_SO_4_, 0.65 mM MgSO_4_.7H_2_O, 0.1 mM KH_2_PO_4_, 10 µM H_3_BO_3_, 0,1 µM MnSO_4_, 50 nM CuSO_4_, 5 nM (NH_4_)_6_Mo_7_O_24_ and 50 µM Fe-EDTA. ZnSO_4_ was added to a final concentration of 50 nM in control conditions, or at indicated concentrations (50 µM or 100 µM). Plates were kept at 22 ºC with 16 h of light/ 8 h of dark in growth chambers until the time of analysis.

### Preparation of samples and mapping by two-dimensional XRF

In all experiments, we compared the detached 7th true leaf of each plant cultivated under the axenic conditions described above. 2D elemental mapping (between 7 and 2 µm steps) was conducted on fresh, unfixed leaf samples. Dehydration was minimized by sealing the leaf sample between two layers of Kapton tape, and the duration of mapping was restricted to less than 2 h. Samples did not show clear signs of dehydration, such as change in size or dried margins. Elemental maps were collected at beamline BL2-3 of the Stanford Synchrotron Radiation Lightsource (SSRL) as described^60^. Beam line 2–3 uses a water-cooled double crystal monochromator with either a Si(220) or Si(111) crystal and a Vortex single element detector. The beam was focused using a Pt-coated Kirkpatrick-Baez mirror pair (Xradia Inc.) and tuned to 11 keV. All maps were collected from the 7th true leaf. Elemental mapping was performed in 7 μm steps with a 50 ms dwell time for whole leaf maps, and in 2 µm steps and 50 ms dwell time for trichome 2D mapping. We used only one leaf per genotype for this analysis, so we confirmed our observations with separate pilot experiments conducted at NSLS beamline X26A, in which we observed the same trichome Zn distribution pattern (Supplemental Fig. 1).

The XRF maps were analyzed using Sam's Microanalysis Toolkit^61^ (https://www.sams-xrays.com/smak). To determine total abundance of elements in whole leaf maps and in individual trichomes, user-defined region of interest (ROI) analyses was performed. First, we defined a region at each trichome base, from which Zn counts were summed. The total number of pixels defined in each ROI was also obtained. The number generated contains all Zn within trichomes plus the Zn in the underlying leaf tissue. To subtract the background counts in each trichome ROI, we selected most leaf areas without trichomes and obtained the mean count per pixel in the leaf. The mean count per pixel in leaf was multiplied by the number of pixels in each ROI and subtracted from the total Zn per trichome value. The percentage of elements in trichomes was calculated by the sum of total abundance in each trichome divided by total abundance in whole leaf map.

### Statistical analyses

When appropriate, data were subjected to ANOVA and means were compared by the Tukey HSD or Student’s t-test using the JMP 10.0 for Mac (SAS Inc., USA).

## Supplementary Information

Supplementary Information
