# Supplementary material for: Arabidopsis thaliana zinc accumulation in leaf trichomes is correlated with zinc concentration in leaves
Source: Sci Rep. 2021 Mar 5;11:5278. doi: 10.1038/s41598-021-84508-y (PMC7935932; doi:10.1038/s41598-021-84508-y)
Supplement: Supplementary file 1 — Supplementary Information [file 41598_2021_84508_MOESM1_ESM.pdf]

Arabidopsis thaliana zinc accumulation in leaf trichomes is correlated with zinc concentration in leaves

Felipe K. Ricachenevsky[1,2,3+*], Tracy Punshon[3+], David E. Salt[4], Janette P. Fett[1,2], Mary Lou Guerinot[3*]

[1]Programa de Pós-Graduação em Biologia Celular e Molecular, Centro de Biotecnologia, Universidade Federal do Rio Grande do Sul, Brazil.

[2]Departamento de Botânica, Instituto de Biociências, Universidade Federal do Rio Grande do Sul.

[3]Department of Biological Sciences, Dartmouth College, USA.

[4]Future Food Beacon of Excellence and the School of Biosciences, University of Nottingham, LE12 5RD, UK.

*** Corresponding authors:** Dartmouth College, Department of Biological Sciences, Life Sciences Center, 78 College St. 03755, New Hampshire, United States.

Email: mary.lou.guerinot@dartmouth.edu

Universidade Federal do Rio Grande do Sul, Instituto de Biociências, Departamento de Botânica. Av. Bento Gonçalves, 9500, Porto Alegre, Rio Grande do Sul, Brazil.

Email: felipecruzalta@gmail.com

Phone number: +55 51 3308 3673

[+] These authors contributed equally to this work

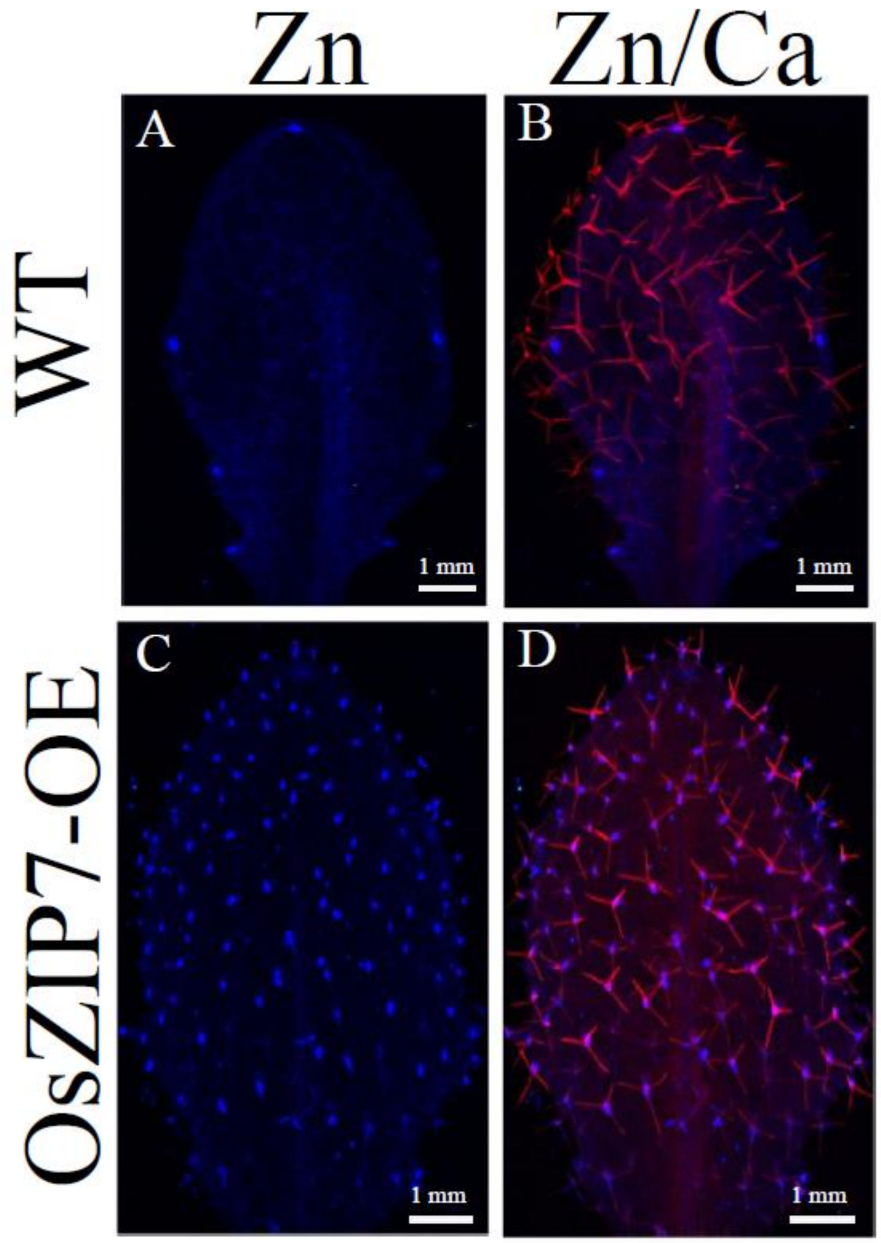

Supplemental Figure 1. Elemental maps of Col-0 (WT) and OsZIP7-OE in leaves of plants grown under 50 nM Zn. Maps show Zn localization (in blue) and Ca (in red).
